# Expanding transformative experience

**DOI:** 10.1111/ejop.12480

**Published:** 2019-10-30

**Authors:** Havi Carel, Ian James Kidd

**Affiliations:** ^1^ Department of Philosophy University of Bristol Bristol UK; ^2^ Department of Philosophy Nottingham University Nottingham UK

## Abstract

We develop a broader, more fine‐grained taxonomy of forms of transformative experience, inspired by the work of L. A. Paul. Our vulnerability to such experiences arises, we argue, due to the vulnerability, dependence, and affliction intrinsic to the human condition. We use this trio to distinguish a variety of positively, negatively, and ambivalently valenced forms of epistemically and/or personally transformative experiences. Moreover, we argue that many transformative experiences can arise gradually and cumulatively, unfolding over the course of longer periods of time.

## 1 INTRODUCTION

The concept of transformative experience (TE), developed by L. A. Paul and fully articulated in her 2014 book of the same title, has recently received much attention from philosophers, including a journal special issue and a book symposium in 2015, an “Author Meets Critics” session at the 2015 Pacific APA, several book reviews, an edited volume currently in preparation, a monograph in preparation, and a number of papers (special issue: *Res Philosophica* 92:2; book symposium: *Philosophy and Phenomenological Research* 91:3; Pettigrew, 2016; 2015; Talbot, 2016; Shupe, 2016; Cappelen & Dever, 2017 Campbell,^2015^).

Such scholarly interest reflects the richness of Paul's guiding conception of how people can change by undergoing a special kind of experience, distinctive by virtue of its capacity to be transformative, in a double sense. First, such experiences are *epistemically transformative*, providing forms or degrees of knowledge and understanding that were previously unavailable and, more importantly, previously inaccessible, insofar as they depend on having the relevant experience—tasting the durian fruit is one of Paul's examples. Second, such experiences are *personally transformative*, fundamentally changing one's values, preferences, desires, and, therefore, transforming one's identity in substantive ways.

Paul argues that such TE challenge certain culturally entrenched conceptions of the nature of human agency, ones that, very broadly, tend to presuppose that our choices are typically appraised and selected against a background characterised by agential autonomy, informed understanding, and situational stability. In the specific case of models of rational decision making on which Paul mainly focuses, agents are conceived as prospectively identifying the possible outcomes of a decision, assigning values to them, and finally weighing those outcomes against her preferences, given their likelihood. Paul argues that this sanguine vision is challenged by the phenomenon of transformative experiences, for these fundamentally change one's values, concerns, and preferences. As a result, the person who contemplates undertaking the experience may be—and is likely to be—quite different to the person who subsequently undergoes it, or the person who emerges at the other side. As Paul (2014) nicely puts it, “[w]e only learn what we need to know after we've done it, and we change ourselves in the process of doing it” (p. 4). In this way, the idea of transformative experience promises to challenge those conceptions of the nature of human agency.

More fully, what Paul develops in her book is the conundrum of having to choose an experience without the knowledge of what it will be like, how we might respond to it, or how it might change us. Such experiences have an intrinsic opacity, insofar as we cannot know in advance what they will be like, or how they will affect our self‐understanding, or even know whether whatever sense we do eventually make of them will be congruent with the sorts of understanding one currently enjoys—for instance, attitudes that I currently find unintelligible may, after one of these experiences, come to seem self‐evident. Paul explains:

The problem is pressing because many of life's big personal decisions are like this: they involve the choice to undergo a dramatically new experience that will change your life in important ways […] But as it turns out […] many of these big decisions involve choices to have experiences that teach us things we cannot know about from any other source but the experience itself. (Paul, 2014, p. 3)

We suggest that further reflection on transformative experience offers a more complex, radical challenge to those conceptions of human agency, such that Paul's challenge can be made broader as we describe below. In order to bring these further challenging dimensions into view, we offer a finer grained taxonomy of types of transformative experience. Moreover, expanding the range of types of transformative experience can help us form connections with similarly complex accounts of human agency, sensitive to the psychological and existential complexities of human experience. An obvious example is existentialism, with its emphases on the ways that judgments, deliberations, and decisions are complicated by “bad faith,” inauthenticity, and other integral features of our “being‐in‐the‐world,” which ensure our efforts to comport ourselves within the world are conditioned by—to quote Heidegger (1996, pp. 169, 179)—the “pre‐given” world of “being‐with‐others,” something unchosen, that is “always, already” in place, into which we are “thrown” (see Cooper, 1999).

We propose, then, that the notion of TE is (a) much broader than has so far been articulated in ways that (b) reveal further, subtler forms of challenge to conceptions of human agency than those focused on by Paul, in ways that also (c) require further attention to the cultural and contextual constraints of the majority of human agents, whose agency is maximally restricted by the limited choices afforded by their social environments or who do not conceive of their agency as the free exercise of choice at all. By doing this, we hope to extend Paul's work towards creating a more pluralistic account of transformative experience that is more faithful to the heterogeneity and variability of human experience, in ways that increase its scope and significance.

Moreover, pluralism of this sort also helps to underscore certain truths about human existence, ones evident in writings by feminist and care ethicists, Buddhist philosophy, and—our focus—the work of Alasdair MacIntyre. Despite their differences, they all regard it as imperative that philosophy centrally affirm the fact that human life is largely characterised by contingency, vulnerability, and lack of control—which we refer to hereafter as “the facts of life.” An important manifestation of these facts is, of course, our ineradicable susceptibility to transformative experiences.

We therefore propose

1. that the concept of TE should be expanded beyond its current link to decision‐theory, with its focus on situations where agents are deliberating whether to freely elect to pursue an experience.
2. a broader taxonomy of types of TE, which includes the *voluntary* cases on which Paul concentrates, but also *involuntary* and *nonvoluntary* experiences, which she briefly mentions but does not discuss (Paul, 2014, p. 16; and cf. Pettigrew unpublished).
3. that this expanded sense of the forms of TE should incorporate a more realistic set of “facts of life” with special pertinence to those experiences: namely, radical contingency, vulnerability to change, and lack of control.
4. that mundane experiences can have a cumulative effect resulting in transformation on a par with that of TE.

The structure of the paper is as follows. Section 2 sets out the “facts of life,” which we believe are pertinent to TE and which may serve as a useful framework for the expanded study of TE. Section 3 offers a broader taxonomy of TE, whereas the fourth examines what kinds of experiences can count as transformative and suggests that everyday experiences can have a cumulative effect that matches that of TE.

## 2 THE FACTS OF LIFE: CONTINGENCY, VULNERABILITY, AND SUBJECTION

Human life takes many forms, but they share certain common features. Often, the features on which philosophers focus are positive—reason, autonomy, moral sophistication, virtue. But there are other, less attractive, features of human life. We are subject to a variety of contingencies—bodily, social, and practical factors that can, and often do, fail us in deep and troubling ways, sometimes suddenly, at other times with slowly unfolding inevitability. As embodied beings, we are destined to injury, illness, and ageing. As social creatures, ones disposed to form communities and engage in shared practices with others, many of us are destined to abuse, exploitation, and oppression. As initiators of projects that aim to give order and meaning to our life, we face the prospect of their failing or going wrong in ways we did not expect and cannot correct.

Gathering these together, we can say that three deep features of human life are *contingency*, *vulnerability*, and *subjection.* They are not periodic features of the lives of certain unfortunate human beings, but universal features of human life as such—they are *existentialia*, in Heidegger's sense of the term of essential features of *Dasein*, of creatures with our “manner of being” (Heidegger, 1996).

Alasdair MacIntyre expresses these features of the human condition:

We human beings are vulnerable to many kinds of affliction and most of us are at some time afflicted by serious ills. How we cope is only in small part up to us. It is most often to others that we owe our survival, let alone our flourishing, as we encounter bodily illness and injury, inadequate nutrition, mental defect and disturbance, and human aggression and neglect. (MacIntyre, 1999, p. 1)

Although not thinking specifically of transformative experiences, MacIntyre is concerned with how we can respond to significant life events, what sorts of virtues or qualities are displayed in these responses, and how an active awareness of these facts of life should transform our thinking about our lives:

These two related sets of facts, those concerning our vulnerabilities and afflictions and those concerning the extent of our dependence on particular others are so evidently of singular importance that it might seem that no account of the human condition whose authors hoped to achieve credibility could avoid giving them a central place. (MacIntyre, 1999, p. 1)

We suggest that these features of human life indicate certain ways of expanding the idea of a transformative experience, by adding forms premised upon these “facts of life.” If our epistemic and practical agency takes place against a complex background of contingency, vulnerability, and subjection, then few of us enjoy anything like optimal conditions for the careful, procedural deliberation and decision that, ideally, human agency requires. Many of the major experiences of the lives of most people are not elected or chosen—we do not *select* them but are *subjected* to them. Some TEs will be chosen after deliberation, but many will not be—natural and social upheaval, accident and trauma, sudden illness, subjection to physical and psychological violence, and so on. In other cases, a person might elect to seek out one type of transformative experience, such as having a child, only to find themselves subjected to another more awful type of transformative experience, such as parental grief at the tragic fate of a stillborn. We appreciate that decision theory offers sophisticated means to address such unpredictable or rare outcomes of decisions. We are not seeking to criticise or engage with decision theory itself. Rather, what we propose to do here is offer an alternative view of human agency and demonstrate how this view leads us to extend the types of TE and the interaction between epistemic and personal transformation.

We thus suggest that human agency is better conceived of as deeply conditioned by complexly dappled sets of circumstances, such as the constant possibility of our subjection to undesired situations, and the absence of optimal conditions for properly epistemically procedural deliberation. These features are evident in many cases of transformative experience, not all of which need to be elected by the agent. As such, we propose expanding the notion of TE, so as to include what we call involuntary and nonvoluntary TEs, in addition to the voluntary TEs that have received attention to date (see Section 3). To develop Paul's headline case of choosing to become a vampire, this is transformative since, prior to being obligingly “turned” by some current member of the undead, one cannot know one's future preferences (whether, once a vampire, I prefer being immortal to being mortal) or the nature of the experience (what it is like to subsist on blood, etc.). But it's also often the case, in vampire novels and films, that people are “turned” against their will when they are attacked by vampires, such as Lucy Westenra in Bram Stoker's novel, *Dracula.* If so, then even with Paul's own examples, we see the possibility for transformative experiences other than the voluntary.

The possibility of transformative experiences outside the category of the voluntary is a consequence of another “fact of life,” namely, the contingent, unchosen character of most of the material and social conditions of the evolution and development of an individual life. Many of these experiences are not, could not, and would not be chosen. Consider some potential major life events—serious accident, chronic illness, separation from a loved one, war, forced migration, poverty, famine, being the victim of a violent crime, suffering severe depression, and the variety of other traumatic experiences that may befall any of us. Note, too, that a person's subjection to these sorts of experiences is shaped by social, economic, geographical, and cultural factors that vary within and across societies. These experiences are of a different *kind* to other sorts of life experience, such as choosing which college to attend, in which neighbourhood to buy a flat, or—Paul's example again—choosing whether to have children.

Take the much discussed situation of making a decision to have a child together with one's partner (e.g., Barnes, 2015; Paul, 2014, 2015a, 2015b; Harman, 2015)—although here, to keep our example simple, we refer to a couple at least one of whom is a woman and who choose to conceive rather than adopt or use surrogacy. Such a situation assumes an egalitarian relationship in which women have as much say as men; the availability and permissibility of forms of contraception; the ability to conceive unproblematically; some minimal resources required to bring up a child; absence of repressive religious or social control of reproductive decisions, and so on. Such biological, social, and practical conditions are highly specific, and only apply to a small percentage of people in the world—roughly, those living in medically advanced and socially liberal developed world societies (although, of course, not only those and not all of those). Many millions of women in today's world, and almost all women historically, did not and do not have reproductive control or have egalitarian relationships or live in a society in which their views are taken into account (cf. Hrdy, 1999).

The current framework also presupposes that *having a child* will be the outcome of a *choice:* that it is an open question whether or not a person (or a couple) *will* have children—something most of the world's 1.2 billion Catholics, for example, will vehemently deny. What this example demonstrates is the *radical specificity* of that particular way of thinking about human lives, action, and agency, and of the contingency of the conditions that must be obtained if the example is to stand. Such deliberative conditions cannot be taken for granted and are largely a particular mode of understanding and enacting one's life and identity.

We claim that the emphasis on choice (see Paul, 2014; Shupe, 2016; Pettigrew, unpublished manuscript) presupposes several things that come under pressure on the MacIntyrean “facts of life” view. First, discussing choices in this way makes an (at least tacit) *assumption of control* over choices and also over the consequences that follow a particular choice. However, as we know, making a choice does not necessarily bring about the desired outcome. Let us return to our couple who have made the choice to become parents and excitedly try to conceive, charting the woman's ovulation cycle, having intercourse at the optimal time, and so on, only to find that one of them is infertile. It's important to amend Paul's discussion of this example by noting that one cannot *decide to have children*; one can only decide to *try* to have children, where success is contingent on a range of factors, many beyond the knowledge and control of the agents. Our choices are often confounded by material and other facts about our abilities and bodily constraints. We can never fully anticipate the outcome of our choices or dictate the neat unfolding of our future subsequent to a certain decision being made.^1^ It is not our claim that decision theory cannot handle such cases, but that they are not the paradigmatic ones within that literature, whereas on the “facts of life” view, they are.

Second, we also wish to reexamine the TE literature's focus on *decision* and *choice.* Paul argues that problems arise, on her account, because, “first, we want to make [a] decision based on [a] phenomenal outcome,” and, second, “if our choice involves an outcome that is epistemically transformative, we cannot know the value of this outcome before we experience it” (Paul, 2015b, p. 10). So, for Paul, making a rational decision means being able to (a) identify the possible outcomes, then (b) assign values to the outcomes, and finally (c) make a rational decision on that basis.

We suggest that many people's lives are so turbulent, fragile, and unstable that this procedure is untenable, both in principle and in practice, for one or more of the following reasons.^2^ First, some people cannot identify the possible outcomes: Their world and their actual and likely position within it is too unstable, as when crossing the border into another country in order to claim asylum, an act that may have multiple outcomes that cannot be securely predicted. Is it better to remain in one's village living in constant fear of violence, or to risk the long and dangerous walk to the border? Of course, it is still possible to act under conditions of radical uncertainty, but the inability to deliberate is itself a harm that merits articulation and amelioration.^3^

Second, some people cannot assign values—how could they, in an unstable world, where their subjection to material and psychological pressures, exacerbated by lack of resources and support, might force rethinking of core commitments or require painful actions formerly considered unconscionable. Sophie, the eponymous protagonist of William Styron's (1979) novel, *Sophie's Choice*, is required by a Nazi doctor to choose which of her two children to send to the gas chambers, on pain of losing both. Sophie “chooses” to send her daughter to death (although her son is murdered later, too), a choice most parents would never feel able to make but which, in her panic, she blurts out and then carries with her for the remainder of her life.^4^

Finally, some people cannot engage in a rational deliberative procedure, if subjection to oppression, violence, and neglect has left them overwhelmed, traumatised, or in some other way cognitively and epistemically compromised. Although they may be free, in some minimal respect, to make choices, they have little or no capacity to reflect on their freedom and choices. As Sartre points out, freedom without a capacity to reflect on it is insufficient, because this severs the relation of freedom to responsibility for the outcomes of one's decisions (Sartre, 1948). We also rely here on the work of critics of “ideal theory” who have issued powerful challenges to conceptions of human agency that presuppose that our capacities are typically in excellent working order and exercised under receptive material and social conditions (cf. Tessman, 2005; Tong and Williams, 2018). Throughout the TE literature, there is a tacit assumption that life contains less contingency than the “facts of life” view suggests. But, as the Stoics remind us, the vagaries of life often descend uninvited, and the only thing that is up to us is how we respond to such events.

Whether at the level of an individual's life or the wider course of societies and cultures, far less of life is the outcome of careful deliberation and decision than is appreciated, not least because reflection on such pervasive contingency can induce a disturbing sense of vulnerability, vividly characterised by various existentialists (see Cooper, 1999, Chapter 8). Karl Jaspers, for instance, speaks of a “metaphysical fear,” arising during what he calls “boundary situations” marked by acute personal challenge, which render null typical values and expectations, forcing one to “decide for oneself how to respond” (Jaspers, 1969). A similarly acute sense of contingency and subjection flow through the writings of Maurice Merleau‐Ponty: a person cannot “choose [themselves] from nothing at all,” in the sense of being able to “transform” their values, commitments, and projects at will. For our reflections and choices always presuppose a “previous acquisition,” like a sense of what matters, something inherited from a surrounding world that we did not choose and over which we have little power (Merleau‐Ponty, 1962, pp. 447, 452, 191 ff). The Buddhist tradition, too, emphasises the “dependent co‐origination” (*paticca‐samutpada*) of things in the world, oneself included. Understanding the deep dependence and contingency that marks human life is an epistemic achievement, and honestly acknowledging it in one's ways of living is a personal and moral achievement—a double feat that makes reflectively responding to the contingency of human life a “Noble Truth.”

We suggest an analogy to a certain view of evolutionary theory: the forces of natural selection act on organisms, such that better adapted organisms are continuously selected, but there is also a dominant element of contingency (e.g., a meteorite strike terminating the reign of dinosaurs) that may lead some species to extinction not because they were maladapted but because of brute contingency (Gould, 1989). Similarly, other species may succeed largely due to chance and not evolutionary forces or adaptation. So, some aspects of some lives are shaped by choice, but contingency plays a powerful role in the shaping of lives.

The major life experiences we call attention to here as TE are ones that are not, to date, discussed much in the TE literature (but see Pettigrew unpublished and Paul and Healy 2018). These are TEs that are negative, sprung upon us, and feared, even if we cannot avoid many of them. They are not captured by a voluntaristic and robustly active sense of agency of the sort presupposed by the debate so far. This is for several reasons. First, no reasonable person would willingly choose to undergo these experiences—for instance, to become a victim of violent crime, or to become seriously ill. These are highly negative experiences that may, of course, accrue some goods as a secondary outcome (Kidd, 2012; Carel, 2016a, Chapter 6), but could not be desired by any informed competent person. Second, these sorts of experiences are not of a sort that people are *invited* to consider, contemplate, and choose to have. Trauma victims are victims in part because they have the experience forced upon them: They are *subjected* to the traumatic experience—a main reason trauma is so awful. These experiences—which sadly form a large part of too many human lives —are thus *de facto* excluded from Paul's account.

This theme is familiar from literature—Oedipus chose not to kill his father or sleep with his mother, but in his attempts to avoid these events ended up killing his father and sleeping with his mother. This is a deep lesson depicted, argues Martha Nussbaum, by ancient Greek tragedy, which shows how people can be “ruined” by “things that just happen to them,” “circumstances whose origin does not lie with them,” and the myriad of “things that they do not control,” due to the “ungoverned contingency [of] social life” (Nussbaum, 2001, pp. 25, 89).

Although Oedipus is a dramatic example, in both senses of the term, it does illustrate a general truth about human life: in any decision‐making procedure made in the thick of life, the decision could be thwarted, twisted, or otherwise frustrated by a variety of factors and forces, many of them beyond our knowledge, understanding, and control. In the words of Robert Burns, in a 1785 poem to a mouse whose nest he accidentally overturned with his plough: “The best laid schemes o' Mice an' Men/Gang aft a‐gley”—often go awry.^5^

Of course, one can respond that decision theory allows for such downstream outcomes to be considered and weighed accordingly. Our point is not that decision theory cannot handle such cases, but that the framework considering rational choice tends to downplay contextual, emotional, situational, and nonrational aspects of choice making in such cases. Here, we provide an account of these aspects and use this to further develop the TE framework.

A consciously elected life‐changing decision may turn out to be life changing, but not at all in the ways intended. This need not be necessarily due to any error or deficiency on the deliberator' part, but simply as an unavoidable consequence of the complexity and contingency of the world. As Epictetus tells us in the opening sentence of the *Encheiridion*, “There are things which are within our power, and there are things which are beyond our power.”^6^ We might add to these things that we wrongly think are within our power, things we wrongly think beyond our power, and also those cases where our power over things shifts suddenly—our bodies, for instance, which can be permanently damaged by an injury that takes only seconds to occur. As a general rule, people tend to exaggerate the number of things that fall under the former category of “things within our power” (see Benatar, 2017, Chapter 4).

We claim that TE is important because it is transformative, not because it is the product of rational deliberation. We therefore claim that in order to see the concept in its full glory, we need to replace the current backdrop assumptions with a MacIntyerean framework, within which TE of different varieties—voluntary, nonvoluntary, and involuntary—can be fully explored. We also claim that the current TE literature's emphasis on choice is not a good reflection of reality, because human life is much more constrained by vulnerability, affliction, and dependency, than the decision theoretic framework used in this literature allows us to see.

In addition, the experiences that the TE literature analyses are overwhelmingly ones that are positively valenced.^7^ The decision to try for a baby is, in the majority of cases, an occasion for congratulations. But contrast this with cases of TE that are *ambivalent* or just plain *negative.* An ambivalent experience might be the decision to go on a cardiopulmonary transplant waiting list. The waiting times are uncertain, the psychological stress enormous, and the outcomes range from mild success (good quality of life for 5–10 years) to death at surgery. The surgery can be complex, complications are common, recovery is difficult and painful, and rejection and side effects from the copious medications are unavoidable. The person with, say, end stage cardiac disease may make a choice to go on the transplant waiting list, but that choice is an option of last resort and therefore not a full choice. To frame such a decision as choice in the same sense as in the proposition “I chose to live in Manchester” is misleading. It is the only choice other than death. It is therefore not a genuine choice between two or more valid options but a sole choice, the alternative to which is death.^8^

In this kind of situation, the decision to undergo that potentially transformative experience is by no means purely positive, as would the be decision to try a new fruit or try to become a parent, nor is it a choice between two alternative life courses, because in one prong, death is the only outcome. Another feature of such a decision is that it is *time‐sensitive*, insofar as it cannot be delayed or deferred in the way that the decisions Paul discusses as paradigmatic can be. So, the little choice that can be said to be exercised under such conditions has to be rushed through, or else it expires (cf. Carel, 2018, Chapter 5). In other words, under such weighty constraints, it is not clear that this decision qualifies as a choice at all. But it is nonetheless transformative.

We therefore suggest that the concept of a TE should be expanded to capture cases where the experience (a) results from *subjection* rather than *election* and (b) not one that a reasonable person could elect to undergo. In the next section, we develop a new taxonomy of TE that includes such cases. We suggest that a broader conception of the range of types of transformative experiences is needed, which is less vulnerable to the particularities and contingencies of specific cases and is less closely aligned with decision making and choice. Many transformative experiences are not elected and are not related to decision‐making in a significant sense. We now turn to those.

## 3 TOWARDS A BROADER TAXONOMY OF TRANSFORMATIVE EXPERIENCES

We propose three types of TE. Paul describes one, which we will call *voluntary* TE. We add *involuntary* and *nonvoluntary* TE to create a broader taxonomy, more typical of human life, and one that takes into account the “facts of life” discussed above. All three types, we suggest, can be genuinely transformative. Consider the following examples:

*Nonvoluntary*: at the age of 24, Primo Levi was arrested in Italy and sent to the Nazi concentration camp at Auschwitz on the German‐Polish border, where he survived for 22 months. The experience changed his life course and left an indelible mark on Levi. Thus, the experience meets Paul's dual criteria: It was epistemically transformative, as it taught Levi what the experience of starvation, imprisonment, torture, cruelty, total lack of control, severe illness, deprivation, and degradation are. It was also personally transformative, as he himself says: “I do not and cannot know what I would be today if I had not been in the camp [...] this would be, precisely, a case of describing a future that never took place” (Levi, 1979, p. 397). Clearly, Levi's experience was transformative and one that no one would ever choose, although Levi evaluates it, remarkably, at least partially positively, that “in its totality this past has made me richer and surer” (Levi, 1979, p. 398).
*Involuntary*: I see a young child run into the path of an oncoming car. Instinctively, I rush into the road, pick up the child, but the car hits me. The child survives unharmed, but I am severely injured, losing the ability to walk. In this case, I am causally responsible for the outcome, but did not intend it, since I acted instinctively. The experience is also transformative: my new disability changes who I am, my future preferences, and what I know about myself and life.^9^ I would not have chosen to become disabled, but the choice to save the child was taken by me. I am transformed, but through an unintended consequence of a choice I make.

These examples show that nonvoluntary and involuntary TEs are quite conceivable. Although we give two dramatic examples, more mundane ones are available. That being so, we suggest that to date, the TE literature only captured *voluntary TE.* Early in her book, Paul mentions negative and involuntary experiences, including “experiencing a horrific physical attack,” “having a traumatic accident,” “undergoing major surgery,” “experiencing the death of a parent,” and “experiencing the death of a child” (Paul, 2014, p. 16). Throughout the book, though, her discussion focuses on experiences that can be and commonly are chosen, like the decision to try for a child.

Common to those voluntary TEs is a particular type of attitude, one that assumes various optimality conditions, including autonomy, freedom to choose, noncoercion, availability of resources, and having time for deliberation. But these conditions do not always obtain—either optimally, sufficiently, or even minimally—and in the cases where they do not there is space for nonvoluntary and involuntary types of TE.

We therefore suggest that many, if not most, types of TE are *involuntary*, imposed upon us by the contingencies of life. As described above, many of the situations in which people find themselves (admittedly, not the likely readership of this paper) are ones that no one would choose. And yet, they can be and often are the rule rather than the exception. For example, the vast majority of us will die of a serious illness, and yet it is difficult, if not impossible, to imagine that a reasonable person, even if in an epistemically ideal situation, could *want* to become seriously ill. Such contingencies are not the rare and unusual cases such as the one of Oedipus described above, but commonplace and a fundamental experiential currency through which we live. It may be helpful to take a life‐cycle point of view here, which includes the different stages of life, from infancy and dependence, through childhood, adulthood and then old age (and eventually dependence again) to see how common these contingencies are. We suggest that this life cycle perspective can reveal susceptibility to TE as belonging to ordinary lives and to ordinary modes of experiencing (cf. Carel, 2016b).

Paul (2015b) claims that “choosing to undergo an epistemically transformative experience” introduces “a deep subjective unpredictability about the future” (p. 7). We suggest that “deep subjective unpredictability” is often experienced by many people as a fact of their *past* and their *present*—indeed, of their life as a whole, and not just as a highly specific issue pertaining to decisions about the future.^10^ Consider the millions of displaced people, refugees, or those living in extreme poverty. Their unpredictability is not subjective, but situational, and this gives us an indication of the extent to which Paul's account can be helpfully supplanted to include an analysis of situational unpredictability. The sense of ownership, indeed authorship, and authority over one's life is a requirement Paul takes for granted her discussions of TE and moreover is essential for her construal of TE. We suggest expanding the focus so as to include cases that do not conform to this paradigm.

Finally, we would like to point out a set of experiences that come under the umbrella term “choice under coercion,” returning to *Sophie's Choice* (Styron, 1979). The pivotal moment of so‐called choice was certainly transformative for Sophie:

She could not believe any of this. She could not believe that she was now kneeling on the hurtful, abrading concrete, drawing her children towards her so smotheringly tight that she felt that their flesh might be engrafted to hers even through layers of clothes […] “Don't make me choose,” she heard herself plead in a whisper, “I can't choose.” (Styron, 1979, pp. 594–595)

We suggest a double expansion of Paul's notion of choice. First, many common and exemplary types of TEs, such as those arising from serious illness, are not the outcome, and could not conceivably be the outcome, of a rational choice. Second, a coerced choice may be transformative even when devoid of all characteristics of choice (it is not free, minimally constrained, made positively, etc.). In short, coercive and unchosen situations may be transformative.

Let us continue to develop our taxonomy, on the basis of bivalent notions of epistemic transformation and personal transformation. We suggest that each of these modes of transformation can be either positively or negatively valenced, such that TEs could take one of four general forms:

1. *Positive epistemic transformation, positive personal* *transformation*—A paradigmatic example here is having a successful long‐term relationship. The epistemic gains are clear—one learns the meaning of a deep, loving bond, one becomes more calm and confident because of their trust in the other's love, one experiences the world as more benevolent, and so on. The personal gains are also positive and mirror the epistemic gains. One is changed in the ways described above to become a more loving, generous, open person. This is a clear example in which both the epistemic and the personal gains are mutually reinforcing and harmonious.
2. *Positive epistemic transformation, negative personal* *transformation*—Here, one learns new things by having a new experience, but the personal transformation is negative. Our example here is having a child and finding that one is a poor, emotionally stunted parent. Here, the epistemic gains are substantial. The parent finds out that she is impatient, easily bored by infants, and that she less giving than she thought. She now knows more about herself and her life than previously. However, the personal transformation is negative—she becomes a bitter and guilt‐ridden parent who feels deeply regretful about the decision to become a parent. She is not a good parent and her values and preferences reflect a desire to be away from her children, giving rise to deep ambivalence and pain.
3. *Negative epistemic transformation, positive personal* *transformation*—The Christian philosophers Thomas Aquinas and Blaise Pascal both underwent profound religious experiences late in their life. As a consequence, both repudiated their earlier philosophical work, with Aquinas judging his monumental contributions to Christian philosophical theology as “straw” (quoted in Caputo, 1982, p. 253). Both men regarded their religious experiences as profoundly positively transformative, not least because the result, from their perspectives, was a deeper and more authentic appreciation of God and their relationship to him. But there was a strongly negative epistemic transformation—major intellectual projects were abandoned, the knowledge and understanding they earlier produced were renounced, and their epistemic stance shifted from one of robust cognitive enquiry to quiescent meditation on—in Pascal's words—“the vanity of philosophic lives,” of lives that are devoted to theoretical curiosity, including his own earlier mathematical and scientific work (*Pensées* [Lafuma] §§61, 67, 152).
4. *Negative epistemic transformation, negative personal* *transformation*—The person suffering from dementia knows less and is less aware of her surroundings. She loses language and the ability to know where she is. She is confused and behaves erratically due to her neurodegenerative impairment. This is also a tragically negative personal transformation of a most radical sort. The past person is erased and the new minimal person is reduced to the remains of the once vibrant person.

Naturally, certain experiences will be too complex or indeterminate to be so easily classifiable, and we further suggest that there are ambivalent transformative experiences, where it is not easy to see whether the transformation was positive or negative. We take Options 1 and 4 to be uncontroversial. Option 1 captures the cases Paul takes to be paradigmatic—one learns something new and is also personally transformed by the knowledge, both positively. A successful long‐term relationship or a good experience of parenting fall under this category. Option 4 is also uncontroversial—take the above example of dementia patient who forgets increasing amounts of information as her disease progresses and also becomes existentially and personally diminished. Both types of transformation are negative.

But consider the cases in which the transformation is valenced differently—when one is positive and the other negative. Take Option 3. In this option, one loses knowledge, curiosity, and certain forms of epistemic motivation, as described by Aquinas and Pascal. In such cases, epistemic diminishment results in personal transformation (religious conversion) that is deeply positive for the individuals involved. Pascal offers the clearest testimony when describing how certain epistemic losses entailed positive personal transformations. Consider *Pensées* [Lafuma] §§526 and 527 ):

The knowledge of God without that of man's misery causes pride. The knowledge of man's misery without that of God causes despair. The knowledge of Jesus Christ constitutes the middle course, because in Him we find both God and our misery.

Jesus Christ is a God whom we approach without pride, and before whom we humble ourselves without despair.

After his religious experience—the “night of fire” recorded in *Pensées* (Lafuma) §913—Pascal came to regard the redemption of sinful, “wretched” human beings through Jesus Christ as the supreme human good. Attainment of that good requires, among other things, a humbling of our pride, the vice that does more than any other to close a person off to Christian faith. A loss of hubristic confidence in human reason is therefore essential to the attainment of that humility that enables the cultivation of faith; so, a loss of epistemic confidence is a step on the way to profound personal transformation. This case is much less easily understandable to us moderns. Is forsaking intellectual activity in order to gain emotional and spiritual calm an overall positive transformation?

And finally, for Option 2, consider again the earlier quotation from Primo Levi. During his imprisonment and torture, Levi learned everything there is to know about cruelty and degradation. He has certainly acquired deep knowledge of aspects of human existence he would not have known otherwise, so his experiences in the concentration camp were clearly epistemically transformative. He was also personally transformed by those experiences, but in ways that were deeply negative. Levi learned about the limitlessness of human evil—a lesson he surely would have been better off without.

We suggest expanding the account of TE by considering that an experience could be positively transformative in one respect, but negatively transformative in another—that, for instance, an experience could be positively epistemically transformative but negatively personally transformative, or vice versa. Many human experiences, and this includes transformative experiences, are mixed.

We further suggest that epistemic transformation can consist of more than acquiring new knowledge. In other words, there is more to us, epistemically, than what we believe and what we know. The “more” can be our epistemic dispositions, virtues, stances, or psychologies: all of these can fundamentally change in ways we want to say are genuinely epistemically transformative. Fricker (2007, p. 49) offers the specific case of loss of epistemic confidence in an agent's beliefs and capacities through subjection to repeated experiences of injustice and oppression. van Fraassen (2002, p. 155) describes the more expansive epistemic transformation that occurs when a person shifts epistemic stances; for instance, from one marked by a sense of the “exclusive sufficiency” of science, to one of “abiding wonder” at a world that one regards as necessarily too complex or mysterious to admit of exhaustive description by scientific theory. In these cases, an epistemic change has taken place, one that goes beyond changes in one's stock of beliefs or of knowledge. When one shifts between the two stances he describes, what changes is one's entire epistemic comportment.

A further comment is in place about what we mean by negative epistemic transformation: This can be an experience that deprives a person of certain types of knowledge, belief, certainty, or confidence. It could also be a loss of epistemic innocence, often with a deep realisation that, once lost, such confidence could never be restored (Ratcliffe, Rudell, & Smith, 2014). The negative valence is not limited to a loss of knowledge (as in the dementia case) but a loss of other epistemic attitudes or contents (e.g., beliefs or sense of certainty).

Although this is a schematic typology, it does indicate the many forms that a transformative experience can take. In ideal cases, an experience will be positively epistemically and personally transformative. But other experiences might be positively epistemically transformative and have either no significant personally transformative effects, or negative effects. Other experiences might be, at least for some time, ambivalent, with a person being unsure how to valence the experience. The complexity and diversity of human life ensures that all of these possibilities can, have, and will be realised in the course of human life, and that this is a fact that the concept of a transformative experience ought to be expanded to acknowledge.

## 4 TRANSFORMATIVE EXPERIENCES

What counts as a transformative experience? Paul defines TE as being epistemically and personally transformative. But how big need a transformation be in order to count as transformative? In this section, we try to shed light on this question. Two issues drive this section: First, do the two conditions need to be met minimally or maximally? What are the boundaries of epistemic and personal transformation? Second, can cumulative changes that amount to TE serve as a descriptive tool for human life?

Let us begin with the first issue. On first blush, it seems that almost any acquisition of new knowledge or change in epistemic state could count as epistemically transformative, depending on how fine‐grained one requires the definition of “change” to be. To take Paul's example, tasting a new fruit, the durian fruit, gives one new information, namely, what the fruit tastes like. On Paul's account, new information has been provided and therefore the experience is epistemically transformative. As we understand Paul, there is no minimal requirement other than the acquisition of some information that was not available to the agent prior to the experience. We call this the minimalist reading of TE and we take it that this reading does not conflict with Paul's position.

On this minimalist reading, every day we gain new knowledge although this is often minimal, trivial, or tacit. If we had not been gaining knowledge, we would not be changing and developing into adults and we could not claim to have any acquired skills. For example, we can gain wisdom as we age. Cicero describes old age as “a tranquil and serene evening,” and moreover one which has “its own appropriate weapons” (Cicero, 1960, pp. 216–218). Montaigne reluctantly admits that “it may well be that (for those who make good use of their time) knowledge and experience grow with the years,” although other qualities “droop and fade” (de Montaigne, 1991, p. 122). We can acquire traits such as increased tolerance, patience, reduced aggression and excitability, and better judgement.

At least in part, these changes are the result of experiences that transform us. But can these be thought of as TE? We suggest that at least some of them can because they teach us something new we could not have learned otherwise—for example, what it means to age and how slow changes in our embodied situation can affect our existential awareness of our mortality—and that therefore many daily and unexceptional experiences are epistemically transformative.

Similarly, almost any change in one's preferences, desires, values, or goals can count as personally transformative, depending on how fine‐grained we require the account to be. Every day our values and preferences are updated, even if only minimally. Every day we change a bit (or a lot) in the kinds of preferences, goals, and desires we have. Again, otherwise we would not make the transition from childhood to adulthood, and on to older age. We become better planners, with more realistic goals; we become better at recognising and controlling our desires, we have increasingly mature preferences and values, and so on. Again, at least partly, these changes are the result of experiences that transform us. These too, we suggest, amount to personal transformation—not singularly but cumulatively.

We thus have both components of TE in place—epistemic transformation, in virtue of having new experiences every day (even if the experience is one of repetition and tedium), and personal transformation, in virtue of updating our preferences, desires, goals, and values, even if minimally.

An objection may be that the kind of transformation that Paul is trying to capture in her book is a deep, significant, and unexpected transformation. But if we consider the cumulative effect of small changes, we can see how TE can arise from a set of mundane experiences. In fact, TE can be decoupled from any explicit decision‐making process altogether. As Callard (2018) suggests, “the ‘decision model’ is not the best way to understand rational transformation into, say, a mother, wife, or pilgrim and that these transformations needn't be prefaced by, or even contain, decisions to become the person in question” (p. 63). Therefore, we believe that the scope of TE is broader, to include both dramatic experiences of the sort Paul describes as well as a range of mundane experiences that cumulatively affect change that amounts to TE.

This position can be found, for instance, in classical Chinese philosophy, which focuses less on what one scholar calls “big‐moment ethics,” or “moments of sharp moral decision” (such as decisions about whether to kill in self‐defence), and more on small, everyday actions that are less dramatic, but, arguably, no less deeply transformative (cf. Kupperman, 1999, p. 169). Such moments do occur, of course, and their eruption into our lives is very often transformative, but this truth should not occlude another. Reflecting on Confucian ethics, Olberding (2013) emphasises that “our moral lives are often conducted not in the raw tension of dramatic dilemmas, but in the delicate and subtle interactions of daily intercourse with others” (p. 8). Depending on one's character, life, and circumstances, the ordinary and everyday can be as transformative as the periodic and profound. Radical transformation can result from experiences and activities that are, as Zen Buddhists like to say, “Nothing special.”

One upshot of this is that if we plump for the minimalist reading of TE, as outlined above, TE can be used to describe how people change over time. Mundane experiences count as transformative if their cumulative effects are transformative, just as a plurality of individually incidental microaggressions can, over time, radically distort the development of a person's self‐esteem, identity, and capacities. Take, for example, a White person who lives in a non‐White neighbourhood. They will see and experience many small (and possibly large, but here we focus on the small) instances of residents being dismissed by officials, public services being patchy, or police being heavy‐handed with the neighbourhood youths. Over time, the White resident may start to view such incidents as parts in a bigger picture, as evidence of racism and ones that ought not to be dismissed as mishaps or one‐off events. This is the result of “socialisation effects” that cause our values to become closer to those of those around us (Bardi, Buchanan, Goodwin, Slabu, & Robinson, 2014). On this example, many small and seemingly trivial events add up, such that TE is the result of small, cumulative effects. These are not dramatic experiences such as the ones described in the TE literature to date, but, we suggest, TE needn't be dramatic or large. Paul focuses on one subset of TE—chosen, voluntary, big, and life changing. We suggest that TE can also be mundane, unchosen, nonvoluntary or involuntary, and small (for a similar argument, see Callard, 2018, Chapter 1).

This reveals another important use of TE, to help document how living changes the living, so to speak, or describing change and personal growth over time. Employing mundane, small, everyday experiences as cumulatively being a vehicle for TE can serve as a counterpoint to many more dramatic accounts of change, like ones found in existentialist writings—Sartre's *Nausea*, say, or Camus' *The Stranger.* We suggest that without recognising small cumulative changes as amounting to TE, we would not be able to explain how people grow and change over time without having undergone dramatic experiences. Thus, TE is a useful tool for describing how people's views, beliefs, and preferences change both gradually and suddenly.

## 5 CONCLUSION

The aim of this paper was to reconceptualise TE, to enable the concept to be broader, more attuned to the reality of many people's lives, less restricted to certain forms of life, have a richer taxonomy, and account for both voluntary and involuntary, as well as nonvoluntary TEs, as well as for positive, negative, and mixed TEs. The proposed broader conception of TE can also be useful as an account of growth and change in human life that allows descriptions of cumulative changes to account for such change without the need for dramatic experiences.

We set out four ways to achieve this:

1. Incorporating a more realistic set of “facts of life”—radical contingency, vulnerability to change, and lack of control—to this expanded sense of the forms of transformative experience.
2. Moving TE beyond its current focus on situations where agents are consciously deliberating whether to freely elect to pursue a significant experience.
3. Developing a broader taxonomy of types of transformative experience, which includes the *voluntary* cases on which the TE literature concentrates, as well as *involuntary* and *nonvoluntary* experiences.
4. Introducing the cumulative TE in addition to the dramatic life‐changing TE, in order to explain human growth and change.

We believe that this new, expanded view of TE will provide a broader, more inclusive notion which will therefore be relevant to more lives and to more of life.

## ENDNOTES

1 Again, we are not claiming that decision theory does not have responses to these challenges. What we are interested in is the view of agency and the place of the “facts of life” within discussions about choice.

2 We do not claim that such lives are more or less typical than the ones analysed by Paul, but that there is more diversity in life circumstances than Paul considers in her analysis. Such lives are perhaps atypical for philosophers but are common in developing countries, war zones, nondemocratic regimes, and as seen recently in the euphemistic “family separation” catastrophe in the United States, also in procedurally democratic regimes. Such lives are typical of disenfranchised, poor, and discriminated against individuals and groups everywhere.

3 We are grateful to Richard Pettigrew for pointing out to us the usefulness of evaluative decision theory to diagnosing this particular harm.

4 This is an example of what Ruth Chang (2015) calls “choice‐based” transformation, as opposed to “event‐based” transformations discussed by Paul. In “choice‐based” transformation it is the making of the choice itself that is transformative, regardless of its consequences.

5
http://www.robertburns.org/works/75.shtml (accessed July 13, 2018).

6
http://www.gutenberg.org/files/45109/45109-h/45109-h.htm (accessed 30/11/16).

7 For a broader range of nonvoluntary and involuntary TEs sees Richard Pettigrew, *Choosing for Changing Selves* (unpublished manuscript, p. 184).

8 Rachel McKinnon makes a similar point about the decision to undergo gender reassignment. As she puts it: “some trans people's experiences of transition as the only option other than suicide (or a life of extreme unhappiness) shows us that in contexts such as these, choosing to undertake a transformative experience becomes rational” (McKinnon, 2015, pp. 424–425). However, we claim that this does not make the decision rational but that it is not a decision in the full sense at all.

9 For an extreme account of such transformation by disability, see Jean‐Dominic Bauby's account of his stroke and the resulting “locked‐in syndrome” he suffered, described in his memoir, *The Diving Bell and the Butterfly*, originally published in 1997 (English translation 1998, Vintage Books).

10 This point is discussed in detail by Richard Pettigrew in his forthcoming *Choosing for Changing Selves*, see especially Chapter 12.
